# High spatial and temporal variation in biomass composition of the novel aquaculture target *Ecklonia radiata*

**DOI:** 10.1007/s10811-023-02969-2

**Published:** 2023-04-28

**Authors:** Jacob Nepper-Davidsen, Christopher R. K. Glasson, Rebecca J. Lawton, Marie Magnusson

**Affiliations:** 1 Coastal Marine Field Station, School of Science, University of Waikato, Tauranga, New Zealand; 2 Environmental Research Institute, The University of Waikato, Tauranga, New Zealand

**Keywords:** Kelp, Seaweed, Phaeophyceae, Biochemistry, Polysaccharides, Phlorotannins, New Zealand

## Abstract

The biomass composition of kelp varies within species both spatially and temporally. However, this variation in biomass quality has not yet been investigated for the native kelp *Ecklonia radiata* within New Zealand, where the kelp is a target for the emerging seaweed aquaculture industry. In this study we quantified spatial and temporal variation in the composition of *E. radiata* biomass, collected from 12 sites around the North Island of New Zealand and from 12 months across a full year at a single site (*n* = 138). High spatial variation was detected for most components, including alginate (range: 16.6 – 22.7% DW, *n* = 12), fucoidan (range: 1.2 – 1.6% DW, *n* = 12), phlorotannins (range: 4.8 – 9.3% DW, *n* = 72), and glucose (range: 9.3 – 22.6% DW, *n* = 12). The biomass composition of *E. radiata* varied significantly among sites but with no clear patterns among regions, indicating that geographic differences were mostly local rather than regional, possibly due to site-specific environmental conditions. Significant temporal variation (measured by positive autocorrelation between months) was detected in the content of lipids, proteins, glucose, guluronic acid, nitrogen, phosphorous, iodine, arsenic, and mercury, and for the mannuronic to guluronic acid (M:G) ratio. Overall, *E. radiata* had comparable biomass composition to that of commercially grown northern hemisphere species but with substantially higher phlorotannin content. These results demonstrate that *E. radiata* could be a viable southern hemisphere alternative for a broad range of commercial applications.

**Supplementary Information:**

The online version contains supplementary material available at 10.1007/s10811-023-02969-2.

## Introduction

The native kelp *Ecklonia radiata* (C.Ag.) J. Agardh is a target species for the emerging seaweed aquaculture industry in both New Zealand and Australia, with broad distribution across New Zealand (Shears and Babcock 2007) and along the temperate and subtropical coasts of Australia (Wernberg et al. 2019). Recent development of hatchery cultivation protocols (Praeger et al. 2022) has facilitated ocean farming trials in New Zealand and fuelled further interest in the aquaculture of this species. The biomass of *E. radiata* contains a range of biomolecules of commercial interest unique to brown seaweed including 1) alginate, a gelling polysaccharide (hydrocolloid) used in food products, pharmaceuticals, textile printing, and cosmetics (Porse and Rudolph 2017), 2) fucoidan, a bioactive sulfated polysaccharide used in nutraceutical and personal health products (Mak et al. 2013; Hsu and Hwang 2019) and, 3) phlorotannins, a group of bioactive phenolic compounds with potential uses in nutraceuticals, cosmetics, and novel biomaterials (Magnusson et al. 2017; Shrestha et al. 2021). Furthermore, *E. radiata* biomass contains laminarin, a storage polysaccharide important for producing fermented commodities such as biostimulants, plant tonics, and food products (Battacharyya et al. 2015), which is currently the biggest market for kelp biomass in New Zealand (Bradly et al. 2021). Other biomass components include proteins, lipids, and minerals which contribute to the nutritional value and suitability of the seaweed biomass for human consumption—a market that accounts for approximately 1/3 of the total value of the global seaweed industry (FAO 2021). Consequently, the commercial value and application of *E. radiata* will depend largely on biomass composition.

The biomass composition of seaweeds varies within species both spatially, due to genotypic variation and differences in environmental conditions between locations, and temporally, due to variation in environmental conditions between seasons (Gosch et al. 2015; Manns et al. 2017; Mata et al. 2017). For instance, spatial variation in phlorotannin content may be correlated to genotypic differences (Honkanen and Jormalainen 2005) and changes in environmental conditions such as salinity, nutrient availability, and herbivory pressure (Targett and Arnold 1998). Similarly, spatial variation in lipid content was linked to genotypic differences in light and nitrogen availability (Gosch et al. 2015), while spatial variation in alginate content may be corelated to wave exposure (Munda 1987; McHugh 2003). Overall, temporal changes in temperature have been identified as a main driver for temporal variation in carbohydrate and mineral content in kelp (compared to salinity and nutrient availability; Manns et al. 2017).

To date, spatial variation in the biomass composition of *E. radiata* in New Zealand has not been investigated. The large environmental variability within the North Island of New Zealand, such as differences in water temperature between north and south (14—21 °C summer temperature; Wijffels et al. 2018) and local differences in nutrient availability from runoff (20-fold differences in nitrate levels between estuarine systems; Plew et al. 2018), may cause morphological and biochemical variation in *E. radiata* (Targett and Arnold 1998; Fowler-Walker et al. 2006; Manns et al. 2017). Furthermore, *E. radiata* shows strong genetic structure and low gene flow within New Zealand (Nepper-Davidsen et al. 2021), potentially resulting in spatial variation in biomass composition between genetically distinct sub-populations (Honkanen and Jormalainen 2005; Gosch et al. 2015). This environmental and genetic differentiation between sites and regions is therefore likely to result in significant spatial differences in the biomass composition of *E. radiata* within New Zealand.

Temporal variation in biomass composition of *E. radiata* in New Zealand has also never been studied. Temporal studies of the alginate content of *E. radiata* from Australia show inconsistent results, with either the highest content found in austral spring and the lowest in austral autumn in the state of Victoria (Stewart et al. 1961) or the lowest content found in austral spring and the highest in austral winter in the state of South Australia (Lorbeer et al. 2017). Additionally, previous studies on temporal variation in the biomass composition of *E. radiata* were conducted with low temporal resolution (e.g. Stewart et al. 1961; Lorbeer et al. 2017) but indicated that location may be a key driver for variation in biomass composition and that temporal patterns may vary between locations (Jennings and Steinberg 1994). These inconsistencies in temporal patterns and the low temporal resolution of previous studies makes it difficult to draw any general conclusions about patterns of temporal variation in biomass composition for *E. radiata* and highlight the need for detailed studies in New Zealand to be undertaken to inform the emerging seaweed aquaculture industry.

The aim of this research was therefore to analyse spatial and temporal changes in the biomass composition of *E. radiata* within the North Island of New Zealand. Specifically, we wanted to assess 1) the spatial and temporal changes in proximate composition (carbohydrates, proteins, lipids and minerals) and specific key components (alginate, fucoidan, laminarin, and phlorotannins), 2) whether spatial changes are local and/or regional, and 3) any relationships between biomass composition and morphology.

## Materials & methods

### Sample collections

**Fig. 1:**
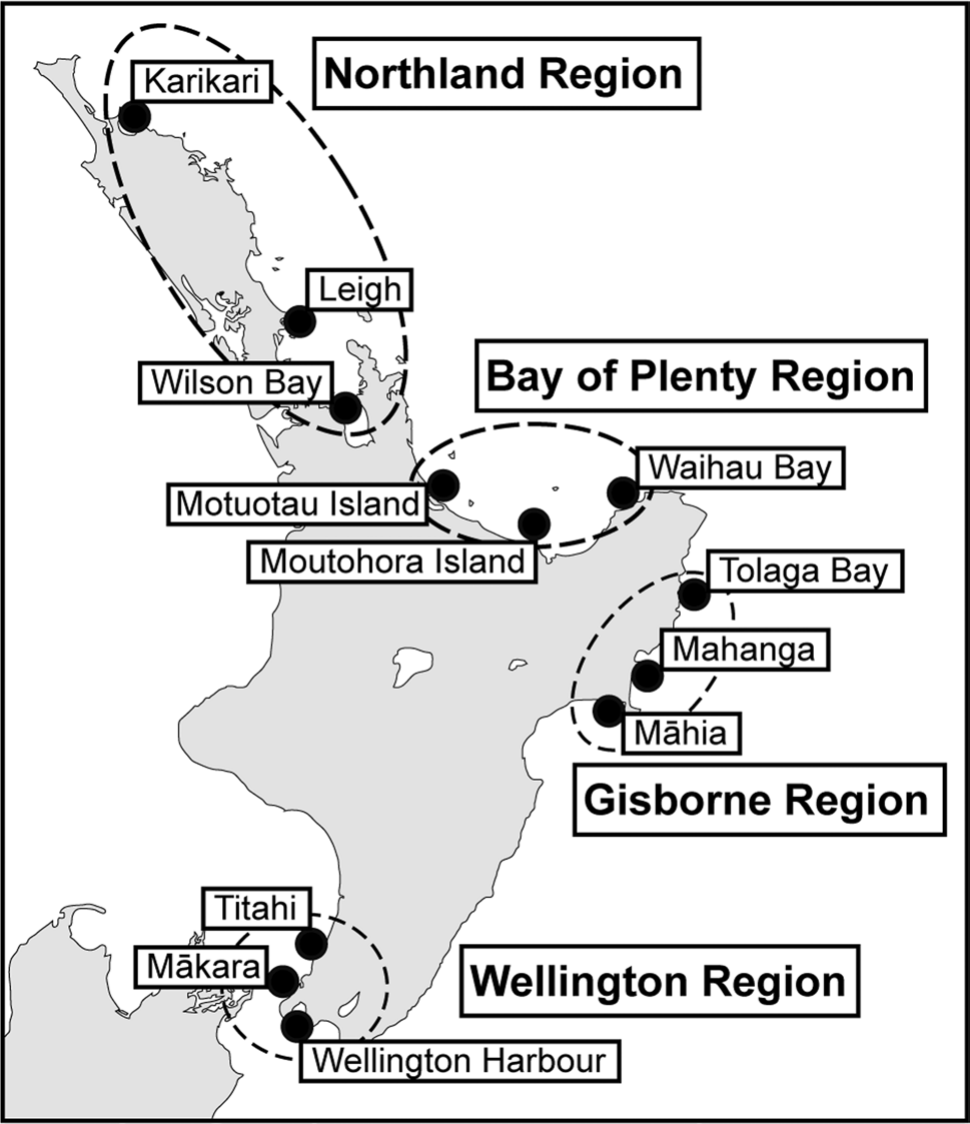
Sampling sites and sampling regions within the North Island of New Zealand

**Fig. 2:**
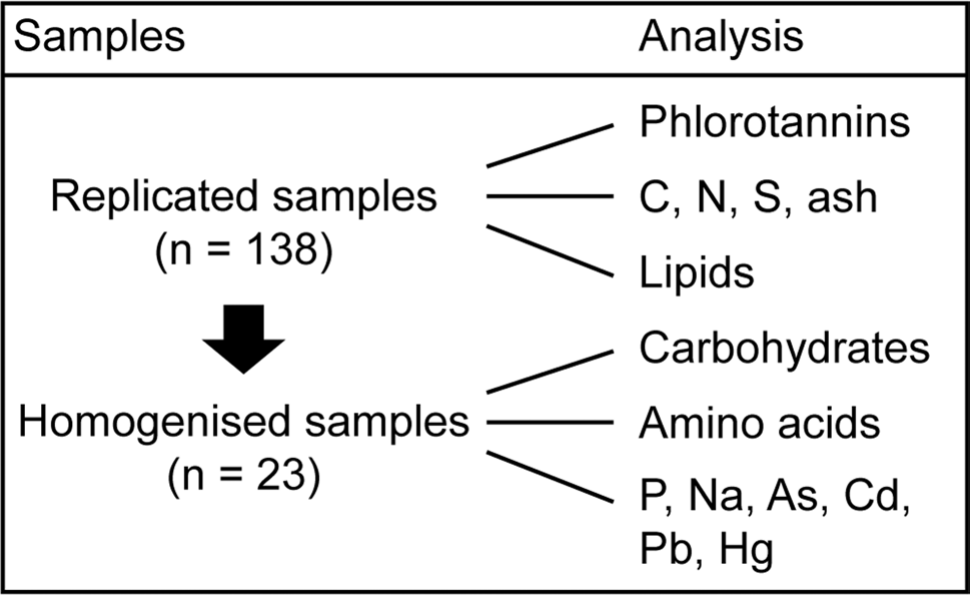
Overview of analyses of biomass components for replicated and homogenised samples. Homogenised samples were made from 2 g DW of each replicated sample, combining the six samples collected at each site and each sampling month

Two groups of *Ecklonia radiata* samples were collected – one spatial and one temporal. For the spatial samples, six whole adult specimens (Stage 3, Kirkman 1984) were collected on a single occasion at 12 different sites within four regions of the North Island of New Zealand, ranging from sub-tropical (Northland region) to temperate (Wellington region) climate (Fig. 1). The spatial samples were collected over a 14 week period from October 2019 to January 2020 (except for the Moutohora Island samples which were collected in March 2020) to minimise temporal variability. For the temporal samples, another six whole adult specimens were collected once every month on 12 occasions during a full year cycle from November 2019 to November 2020 at Motuotau Island in Tauranga (Covid-19 lockdown prevented sampling in April 2020). Samples were collected haphazardly by cutting with a knife just above the holdfast (i.e., samples included thallus and stipe, but no holdfast) while snorkelling on rocky reefs within 2–6 m depth and with > 2 m between each sampled individual. A total of 138 samples (replicated samples) were collected (spatial samples mean weight: 80.7 ± 42.5 SD g dry weight (DW), *n* = 72; temporal samples mean weight: 80.4 ± 39.2 SD g DW, *n* = 72) with the 6 samples collected at Motuotau Island in November 2019 included in both sample groups. Morphology of the stipe, primary lamina, and blades of each specimen were measured in the field after collection (Online resource 1). Samples were stored in separate polyethylene sealable bags and immediately placed on ice for transport back to the laboratory (maximum 48 h). Samples were rinsed in seawater at the collection sites or in 5 µm filtered seawater immediately upon return to the laboratory to remove epiphytes and debris, then weighed (wet weight, WW) and frozen at -20 °C. Samples were freeze-dried to dryness (Buchi, Lyovapor, L-200, Switzerland; 2 mbar, -50 °C), then weighed (DW), milled to < 0.5 mm fine particles (Pulverisette 15 cutting mill, Fritsch GmbH, Germany), and stored in double polyethylene sealable bags with silica gel until further processing. Twenty-four homogenised samples were made by combining subsamples (2.0 g DW) from each of the six samples collected at each site to give 12 spatial samples (one for each site), and from each of the six samples collected each month to give 12 monthly samples (Fig. 2). Mean moisture content following freeze drying was 1.0 ± 0.2 SD % DW for all replicated and homogenised samples (*n* = 162).

### Constituent sugar content

Constituent sugars analysis was adapted and improved from Rozaklis et al. (2002) and Lorbeer et al. (2015). Dried milled biomass samples (10–11 mg weighed to 0.1 mg precision) were hydrolysed in 13 M sulfuric acid (300 µL) for 60 min with stirring, and then diluted with H_2_O (3.6 mL) to 1 M sulfuric acid and heated at 100 °C for an additional 3 h. Following neutralisation with 2 M NaOH (100 µL), hydrolysed samples (100 µL), with added 2-deoxy-D-glucose (40 µL of 10 mg mL^−1^) as internal standard, were derivatised by addition of PMP-derivatising regent (400 µL of 250 mM 1-phenyl-3-methyl-5-pyrazolone with 400 mM NH_3_) and heating at 70 °C over 90 min with constant stirring. This solution was then neutralisation with 0.8 M formic acid (400 µL) and extracted with CHCl_3_ (750 µL) to remove excess PMP. Finally, the supernatant was centrifuged (5 min at 13,000 rpm) and transferred to a 2 mL Shimadzu HPLC vial (Product #: 226–50,512-00) for analysis. Derivatised monosaccharides were quantified using a Shimadzu Prominence LC-20AD (Japan) fitted with a Restek Raptor ARC-18 column (5 µm particle size, 5 µm, 150 × 4.6 mm, Catalogue #: 9,314,565) with an oven temperature of 30 °C and flow rate of 0.8 mL min^−1^. Derivatised monosaccharides were separated after injection of 5 µL of sample by a isocratic/gradient elution with solvent A (0.1 M phosphate buffer at pH 7 in 10% acetonitrile) and solvent B (0.1 M phosphate buffer at pH 7 in 17% acetonitrile); 25% solvent B for 15 min, linear increase from 25 to 100% solvent B over 25 min, 100% solvent B for 15 min, and finally 25% solvent B for 5 min. Derivatised monosaccharides were detected at 254 nm using a photo diode-array detector. The sugars (D-glucose, L-guluronic acid, D-mannuronic acid, L-fucose, D-mannose, D-galactose, D-xylose, L-rhamnose, D-glucuronic acid, and L-arabinose) were identified from their relative retention times compared to the internal standard and quantified using response calibration curves generated from sugar standards with concentrations in the range of 0.01 – 1.0 mg mL^−1^. The % DW and normalised mol % of each anhydro-sugar (as this is the form of sugar present in a polysaccharide) was presented. Total carbohydrates (% DW) were also calculated by deducting the percentages of lipid, protein, phlorotannin, and ash content from 100%.

### Phlorotannin content

Phlorotannin content was estimated as total phenols (Shrestha et al. 2021) which was measured using the Folin-Ciocalteu phenol reagent method (Zhang et al. 2006) adjusted by diluting the extracts to 50% concentration prior to the reaction and by incubating the reactants at 40 °C for 30 min at 100 rpm prior to absorbance reading at 750 nm (SPECTROstar Nano, BMG Labtech, Germany). Phloroglucinol was used as a reference standard, and the results were expressed as phloroglucinol equivalents (PGE, % of DW).

### Amino acid content

Amino acid content was commercially analysed by the Australian Proteome Analysis Facility (Sydney, Australia; anhydro-amino acids) as described in Angell et al. (2014) and included all essential amino acids except cysteine (usually a minor constituent). Protein content was estimated as the sum of the 16 quantified amino acids (Angell et al. 2016) and a nitrogen to protein conversion factor was calculated from the nitrogen and protein content (Protein:*N* = Protein % DW / N % DW).

### Ash and mineral content

Elemental analysis of carbon, hydrogen, nitrogen, and sulfur (CHNS), iodine, and content of ash were determined commercially by OEA labs Ltd (Exeter, UK). Contents of CHNS were determined by gas chromatography coupled to a thermal conductivity detector (GC-TCD), while contents of iodine were determined using ion chromatography (IC) following hydropyrolysis combustion. Content of ash was determined by micro-ashing (Prometheus Kilns Pro1-PRG, Turkey). Further element analysis of phosphorous, potassium, arsenic, cadmium, lead, and mercury (and other minerals: see Online resources 2 and 3) was analysed commercially at the University of Waikato Laboratory (Hamilton, New Zealand) by inductively coupled plasma (ICP) – mass spectroscopy (MS) on homogenised samples (*n* = 24).

### Lipid content

Lipid content was quantified following Folch et al. (1957), as modified and described in Gosch et al. (2012), using dichloromethane and methanol solution at 60 °C for 1 h for extraction, followed by microfiltration of the extract, and addition of 20% (v/v) of a 0.9% sodium chloride solution to separate the lipid fraction. Finally, the solvent was removed from the lipid fraction by heating at 40 °C in a heating block and the weight of the residue was recorded.

### Statistical analysis

Statistical analyses were carried out in R-Studio (Team RStudio 2022) and PRIMER 7 (Clarke and Gorley 2015), and legibility of figures was improved in Adobe Illustrator. Significance of temporal patterns (November 2019 to November 2020) was analysed using Durbin-Watson’s test (lmtest package in R-Studio) which returns a d-value from 0 to 4 where values of 0 to 2 indicate positive autocorrelation, values of 2 indicate no autocorrelation, and values of 2 to 4 indicate negative autocorrelation. Significance of spatial variation was analysed using permutational multivariate analysis of variance (PERMANOVA; as assumptions for analysis of variance (ANOVA) were not generally met) in PRIMER 7 and was calculated for all biomass components measured with replication (i.e. not components quantified from homogenised samples). A nested PERMANOVA design was chosen, testing for differences among regions (fixed factor) and sites nested within regions (random factor), and analyses were run with individual models for each biomass component, using Euclidian distance as distance measurement, and 9,999 unrestricted permutations of raw data. Some significant differences in dispersion were detected among sites and regions using test of homogeneity of dispersion (PERMDISP), however in many cases such differences will not significantly inflate the error rates of PERMANOVA (Anderson et al. 2016). Relationships between morphology and biomass composition were analysed using Pearson correlation coefficients and linear regression models in R-Studio, and correlations were visualised using the ggcorrplot package. Analysis of equal variance between spatial and temporal samples were calculated with Levene’s test (car package in R-studio) using the mean value for each site and each monthly sample. Non-metric MDS was carried out in PRIMER 7 to assess overall patterns in biomass composition among sites within regions and among monthly samples. Data was square root transformed and normalised, and Euclidian distance was used as distance measurement, with 100 restarts and 1 Kruskal fit scheme for each plot.

## Results

### Spatial variation

**Fig. 3:**
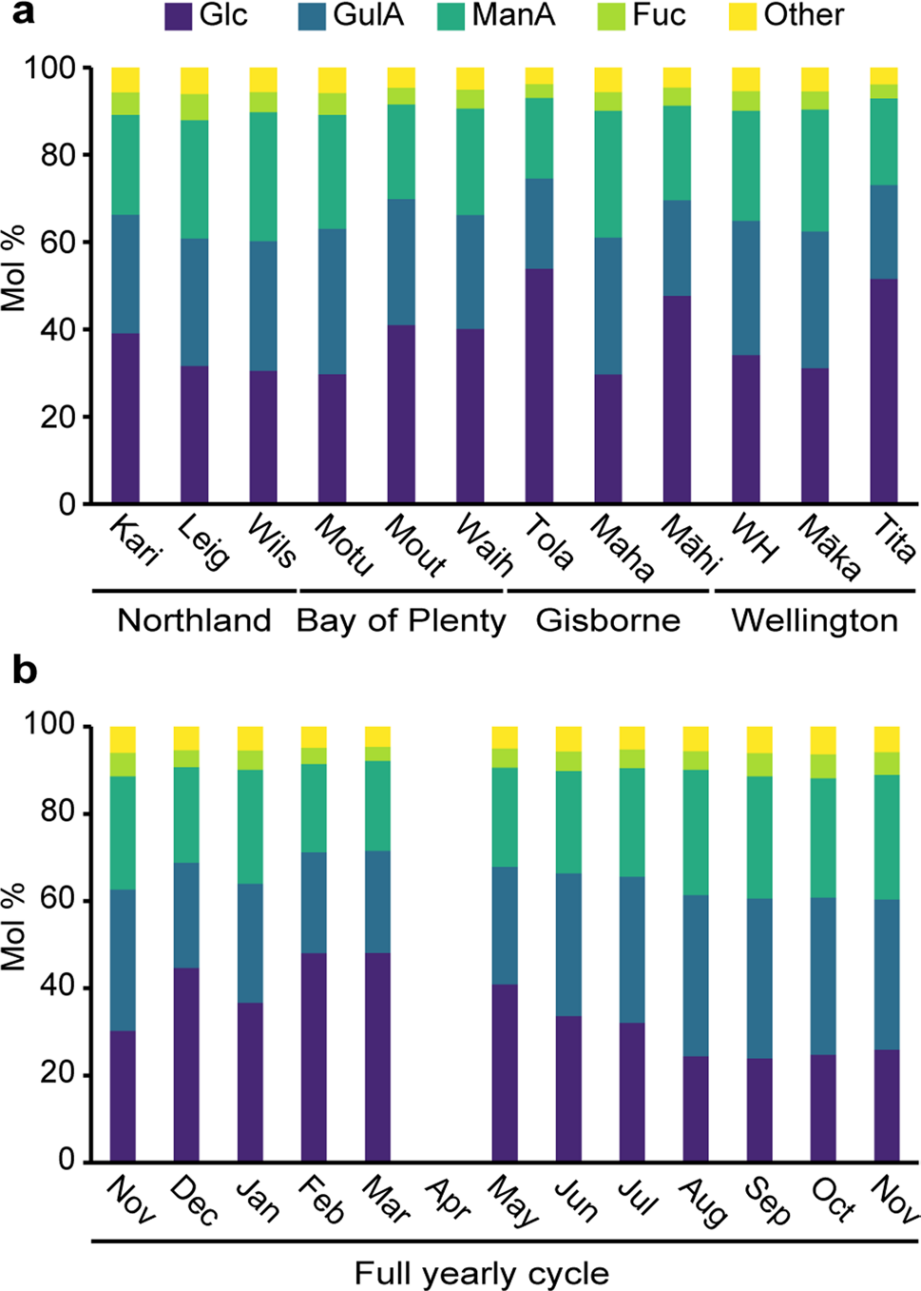
Mole percent (mol %) of quantified sugars: glucose (Glu), guluronic acid (GulA), mannuronic acid (ManA), fucose (Fuc), and other sugars (mannose, galactose, and xylose) of (a) spatial homogenised samples among sites (*n* = 12) and (b) monthly homogenised samples at Motuotau Island (*n* = 12). Site abbreviations are: Karikari (Kari), Leigh (Leig), Wilson Bay (Wils), Motuotau Island (Motu), Moutohora Island (Mout), Waihau Bay (Waih), Tolaga Bay (Tola), Mahanga (Maha), Māhia (Māhi), Wellington Harbour (WH), Mākara Beach (Māka), and Titahi Bay (Tita)

**Fig. 4:**
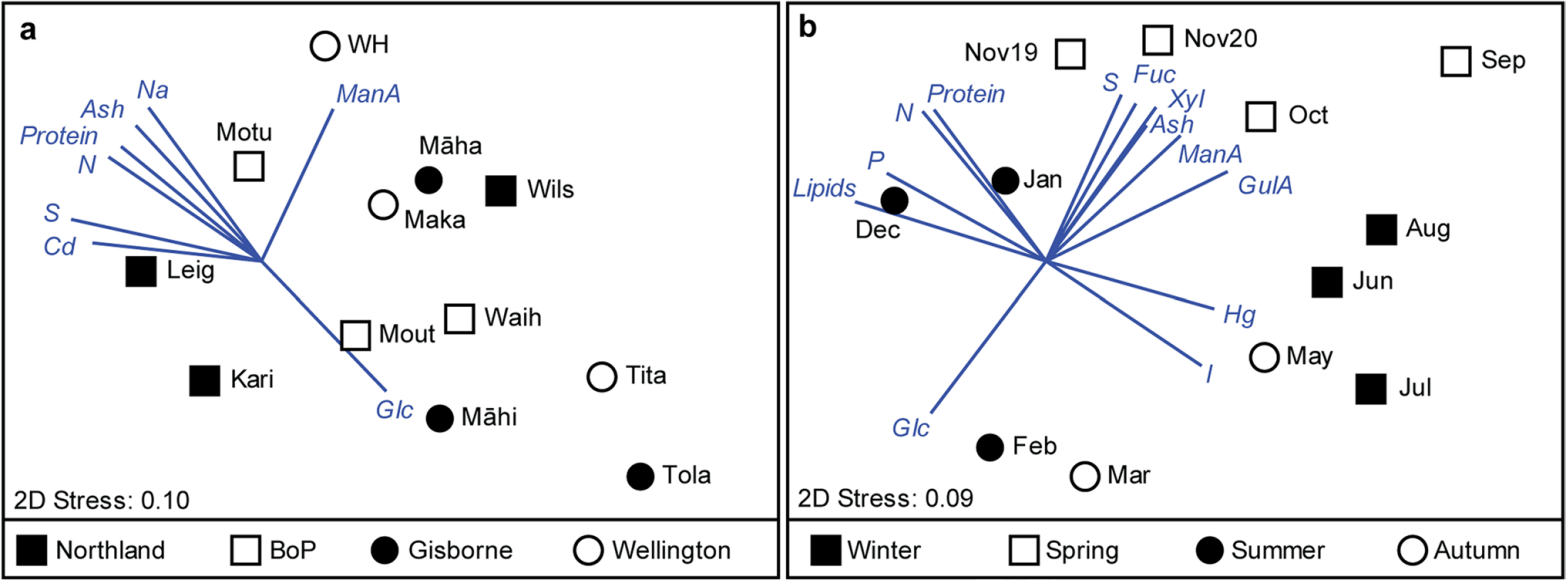
Non-metric multi-dimensional scaling (MDS) of biomass composition of *E. radiata* using all 20 measured biomass components (**a**) among sites within regions (*n* = 12) and (**b**) among monthly samples (*n* = 12). Vectors are shown for biomass components showing > 0.8 correlation (Pearson correlation coefficient). Site and region abbreviations are: Bay of plenty (BoP), Karikari (Kari), Leigh (Leig), Wilson Bay (Wils), Motuotau Island (Motu), Moutohora Island (Mout), Waihau Bay (Waih), Tolaga Bay (Tola), Mahanga (Maha), Māhia (Māhi), Wellington Harbour (WH), Mākara Beach (Māka), and Titahi Bay (Tita). Component abbreviations are: glucose (Glc), guluronic acid (GulA), mannuronic acid (ManA), fucose (Fuc), xylose (Xyl), nitrogen (N), phosphorous (P), potassium (Na), sulfur (S), iodine (I), cadmium (Cd), and mercury (Hg)

High levels of spatial variation between sites were found for most quantified components of *E. radiata* biomass (Table 1). Significant differences between sites were found for all biomass components measured with replication, including total carbohydrates (range: 58.0 – 71.8% DW, *n* = 72), phlorotannins (range: 4.8 – 9.3% DW, *n* = 72), ash (range: 16.2 – 25.1% DW, *n* = 72), nitrogen (range: 0.9 – 1.6% DW, *n* = 72), and sulfur (range: 0.6 – 1.1% DW, *n* = 72; Table 2). Among biomass components not measured with replication (i.e. spatial homogenised samples, *n* = 12; no statistical analysis), glucose content was highly variable among sites (range: 9.3 – 22.6% DW, *n* = 12), whereas guluronic acid (range: 9.0—12.2% DW, *n* = 12), mannuronic acid (range: 7.6 – 10.8% DW, *n* = 12), fucose (range: 1.2 – 1.6% DW, *n* = 12), and the mannuronic to guluronic ratio (M:G ratio, range: 0.75 – 0.99, *n* = 12) were more stable (Fig. 3a). Concentrations of mannose, galactose and xylose were generally low (combined range: 0.3—0.9% DW, *n* = 36), while rhamnose, glucuronic acid, and arabinose were also identified but at concentrations too low for consistent quantification. Proteins also varied substantially (range: 4.6—8.6% DW, *n* = 12), with aspartic acid and glutamic acid being the most variable amino acids, varying two-fold among sites (range: 0.7 -1.2 and 0.8 – 1.7% DW, respectively, *n* = 12; Table 3). Among minerals, a two-fold difference was detected for phosphorous (range: 0.09 – 0.19% DW, *n* = 12) and potassium (range: 2.2 – 4.0% DW, *n* = 12). Non-metric MDS showed limited spatial clustering among sites within regions (Fig. 4a), and similarly, PERMANOVA showed no significant difference between regions (except for lipids; Table 2).

**Table 1:** Spatial variation in biomass content of *E. radiata* collected at 12 sites across the North Island of New Zealand with SD shown in brackets (*n* = 72 for components measured with replication and *n* = 12 for other components). Total carbohydrates (Carbs), phlorotannins (Phlo), proteins, ash, lipids, glucose (Glc), guluronic acid (GulA), mannuronic acid (ManA), fucose (Fuc), mannose (Man), galactose (Gal), xylose (Xyl), nitrogen (N), phosphorus (P), potassium (K), sulfur (S), and iodine (I) are shown as % DW. Arsenic (As), cadmium (Cd), lead (Pb), and mercury (Hg) are shown as ppm DW. M:G shows the mannuronic to guluronic acid ratio, Protein:N shows the nitrogen to protein conversion factor, and C:N shows the carbon to nitrogen ratio. Site abbreviations are: Karikari (Kari), Leigh (Leig), Wilson Bay (Wils), Motuotau Island (Motu), Moutohora Island (Mout), Waihau Bay (Waih), Tolaga Bay (Tola), Mahanga (Maha), Māhia (Māhi), Wellington Harbour (WH), Mākara Beach (Māka), and Titahi Bay (Tita)

	Kari	Leig	Wils	Motu	Mout	Waih	Tola	Māha	Mahi	WH	Māka	Tita	Mean
Carbs _(%)_	60.4 (3.4)	58.6 (2.7)	63.2 (1.5)	58 (2.6)	66.5 (4.0)	67.3 (3.1)	71.5 (2.6)	63.2 (1.5)	71.2 (2.2)	61.5 (3.1)	63.6 (1.5)	71.8 (1.6)	64.7 (4.9)
Phlo _(%)_	9.3 (1.8)	9.1 (1.3)	6.0 (0.7)	8.0 (2.3)	6.3 (0.6)	6.2 (1)	6.0 (1.0)	8.0 (1.4)	5.0 (0.6)	4.8 (0.9)	5.8 (0.8)	5.0 (1.0)	6.6 (1.6)
Protein _(%)_	6.8	6.6	5.3	8.6	5.6	5.2	4.6	6.1	5.5	7.8	6.4	5.2	6.1 (1.2)
Ash _(%)_	22.8 (2.9)	25.1 (1.9)	25 (1.9)	24.7 (2.6)	21.5 (3.7)	21 (2.7)	16.2 (1.3)	21.2 (2.7)	16.6 (1.7)	24.4 (3.2)	23.4 (1.6)	17.5 (1.6)	21.6 (3.3)
Lipids _(%)_	0.7 (0.3)	0.6 (0.3)	0.4 (0.2)	0.7 (0.3)	0.1 (0.1)	0.4 (0.1)	1.7 (0.2)	1.6 (0.1)	1.7 (0.3)	1.4 (0.6)	0.9 (0.4)	0.5 (0.2)	0.9 (0.6)
Glc _(%)_	11.8	9.5	9.8	9.3	15.3	13.5	22.6	10.1	18.8	12.4	10.9	21.5	13.8 (4.5)
GulA _(%)_	9.0	9.6	10.5	11.4	11.8	9.6	9.5	11.7	9.4	12.2	12.0	9.8	10.5 (1.1)
ManA _(%)_	7.6	8.9	10.4	8.9	8.9	9.0	8.5	10.8	9.4	10.0	10.7	9.1	9.4 (0.9)
Fuc _(%)_	1.4	1.6	1.3	1.4	1.3	1.3	1.2	1.3	1.5	1.5	1.3	1.2	1.4 (0.1)
Man _(%)_	0.7	0.8	0.8	0.8	0.8	0.8	0.7	0.9	0.8	0.8	0.9	0.7	0.8 (0.1)
Gal _(%)_	0.6	0.7	0.6	0.6	0.6	0.5	0.6	0.6	0.6	0.7	0.6	0.5	0.6 (0.1)
Xyl _(%)_	0.3	0.3	0.3	0.3	0.3	0.3	0.3	0.3	0.3	0.3	0.3	0.3	0.3 (0.0)
M:G	0.84	0.93	0.99	0.79	0.75	0.93	0.89	0.93	0.99	0.82	0.89	0.92	0.89 (0.07)
N _(%)_	1.4 (0.1)	1.3 (0.1)	1 (0.0)	1.6 (0.1)	1.1 (0.1)	1.1 (0.1)	0.9 (0.1)	1.1 (0.1)	1.1 (0.1)	1.5 (0.1)	1.3 (0.0)	1 (0.1)	1.2 (0.2)
P _(%)_	0.11	0.12	0.17	0.15	0.09	0.11	0.11	0.10	0.11	0.19	0.13	0.13	0.13 (0.03)
K _(%)_	3.3	3.7	3.9	4.0	3.2	2.8	2.2	3.2	2.4	3.8	3.7	2.5	3.2 (0.6)
S _(%)_	1.0 (0.1)	1.1 (0.1)	0.7 (0.0)	1.0 (0.1)	0.9 (0.1)	0.8 (0.1)	0.7 (0.1)	0.9 (0.0)	0.7 (0.1)	0.9 (0.1)	0.8 (0.1)	0.6 (0.1)	0.8 (0.1)
Protein:N	4.9	4.9	5.1	5.3	5.1	4.9	5.3	5.5	5.2	5.1	4.9	5.0	5.1 (0.2)
C:N	23.4	23.7	29.5	19.4	29.1	30.4	39.8	29.4	32.3	20.4	24.3	32.8	27.9 (5.9)
I _(%)_	0.34	0.38	0.40	0.48	0.55	0.44	0.60	0.61	0.46	0.73	0.57	0.66	0.52 (0.12)
As _(ppm)_	50.8	64.4	43.2	54.8	63.5	40.0	40.1	46.4	48.9	48.1	43.2	44.7	49 (8.2)
Cd _(ppm)_	1.3	2.2	0.5	1.9	1.5	0.8	0.8	1.5	1.3	1.2	1.4	0.4	1.2 (0.6)
Pb _(ppm)_	0.3	0.1	0.3	0.2	0.0	0.2	0.7	0.4	0.3	0.5	0.1	0.1	0.3 (0.2)
Hg _(ppm)_	0.00	0.00	0.04	0.02	0.03	0.01	0.03	0.02	0.03	0.01	0.01	0.01	0.02 (0.01)

**Table 2:** Permutational multivariate analysis of variance (PERMANOVA) between spatial samples of *E. radiata* (*n* = 72), testing for differences among regions and sites nested within regions for biomass components measured with replication. Significant differences in dispersion among sites and regions are marked with an asterisk and significant *p*-values are italicised

Source	df	SS	MS	Pseudo-F	P(perm)
Total carbs					
Regions	3	590.4	196.8	1.5	0.282
Sites (nested)	8	1018.4	127.3	18.8	< *0.001*
Res	60	406.8	6.8		
Total	71	2015.5			
Phlorotannins					
Regions	3	75.7	25.2	2.3	0.132
Sites (nested)	8	87.4	10.9	6.1	< *0.001*
Res	60	108.2	1.8		
Total	71	271.3			
Ash					
Regions	3	379.3	126.4	3.1	0.090
Sites (nested)	8	329.2	41.2	5.9	< *0.001*
Res	60	421.2	7.0		
Total	71	1129.7			
Lipids					
Regions	3	16.0	5.3	10.3	*0.007*
Sites (nested)	8	4.2	0.5	5.3	< *0.001*
Res	60	5.8	0.1		
Total	71	26.0			
Nitrogen					
Regions	3	0.9	0.3	1.0	0.465
Sites (nested)	8	2.6	0.3	32.8	< *0.001*
Res	60	0.6	0.0		
Total	71	4.1			
Sulfur					
Regions	3	0.4	0.1	1.7	0.257
Sites (nested)	8	0.7	0.1	10.1	< *0.001*
Res	60	0.5	0.0		
Total	71	1.5

**Table 3:** Amino acids as % DW among spatial samples (all sites, homogenised samples, *n* = 12, top panel) and temporal samples (Motuotau Island only, homogenised samples, *n* = 12, lower panel). Amino acid abbreviations are: histidine (His), serine (Ser), arginine (Arg), glycine (Gly), aspartic acid (Asp), glutamic acid (Glu), threonine (Thr), alanine (Ala), proline (Pro), lysine (Lys), tyrosine (Tyr), methionine (Met), valine (Val), isoleucine (Ile), leucine (Leu), and phenylalanine (Phe). Site abbreviations are: Karikari (Kari), Leigh (Leig), Wilson Bay (Wils), Motuotau Island (Motu), Moutohora Island (Mout), Waihau Bay (Waih), Tolaga Bay (Tola), Mahanga (Maha), Māhia (Māhi), Wellington Harbour (WH), Mākara Beach (Māka), and Titahi Bay (Tita)

	Kari	Leig	Wils	Motu	Mout	Waih	Tola	Maha	Māhi	WH	Māka	Tita	mean (SD)
His	0.14	0.15	0.13	0.18	0.11	0.12	0.11	0.13	0.12	0.17	0.14	0.12	0.13 (0.02)
Ser	0.32	0.33	0.27	0.39	0.26	0.26	0.22	0.29	0.25	0.38	0.31	0.25	0.29 (0.05)
Arg	0.30	0.31	0.25	0.38	0.22	0.23	0.20	0.26	0.23	0.35	0.27	0.23	0.27 (0.05)
Gly	0.35	0.35	0.28	0.41	0.26	0.27	0.23	0.30	0.26	0.40	0.31	0.26	0.31 (0.06)
Asp	0.98	0.93	0.78	1.17	0.81	0.79	0.67	0.95	0.77	1.15	0.97	0.77	0.89 (0.15)
Glu	1.22	1.16	0.81	1.75	1.23	0.84	0.85	1.06	1.14	1.28	1.10	0.89	1.11 (0.25)
Thr	0.35	0.35	0.29	0.45	0.30	0.30	0.25	0.33	0.28	0.44	0.36	0.28	0.33 (0.06)
Ala	0.65	0.58	0.46	0.81	0.49	0.40	0.37	0.49	0.48	0.65	0.55	0.43	0.53 (0.12)
Pro	0.31	0.31	0.25	0.39	0.25	0.25	0.21	0.28	0.23	0.37	0.30	0.24	0.28 (0.05)
Lys	0.38	0.37	0.30	0.47	0.29	0.30	0.26	0.36	0.29	0.48	0.39	0.30	0.35 (0.07)
Tyr	0.14	0.13	0.12	0.18	0.11	0.12	0.11	0.13	0.12	0.20	0.15	0.12	0.13 (0.03)
Met	0.16	0.17	0.14	0.20	0.12	0.13	0.11	0.14	0.13	0.19	0.15	0.13	0.15 (0.03)
Val	0.39	0.39	0.32	0.48	0.30	0.32	0.27	0.36	0.30	0.47	0.37	0.31	0.36 (0.06)
Ile	0.29	0.29	0.24	0.35	0.22	0.22	0.19	0.25	0.22	0.34	0.27	0.22	0.26 (0.05)
Leu	0.49	0.50	0.41	0.60	0.36	0.38	0.34	0.44	0.38	0.57	0.45	0.38	0.44 (0.08)
Phe	0.33	0.33	0.27	0.39	0.24	0.26	0.23	0.30	0.26	0.39	0.31	0.26	0.30 (0.05)
	Nov	Dec	Jan	Feb	Mar	May	Jun	Jul	Aug	Sep	Oct	Nov	mean (SD)
His	0.14	0.15	0.14	0.12	0.11	0.11	0.12	0.12	0.12	0.13	0.15	0.15	0.13 (0.01)
Ser	0.34	0.34	0.34	0.28	0.25	0.27	0.30	0.29	0.32	0.33	0.36	0.35	0.32 (0.03)
Arg	0.32	0.32	0.31	0.24	0.22	0.22	0.25	0.23	0.26	0.27	0.32	0.32	0.27 (0.04)
Gly	0.36	0.36	0.35	0.28	0.26	0.28	0.31	0.29	0.32	0.33	0.37	0.36	0.32 (0.04)
Asp	1.01	1.00	0.98	0.80	0.75	0.80	0.90	0.91	0.97	1.01	1.04	1.00	0.93 (0.10)
Glu	1.53	1.71	1.65	1.57	1.32	1.13	1.08	1.23	1.10	1.04	1.14	1.10	1.30 (0.24)
Thr	0.40	0.38	0.38	0.32	0.29	0.31	0.35	0.35	0.38	0.39	0.41	0.39	0.36 (0.04)
Ala	0.71	0.74	0.70	0.61	0.51	0.50	0.50	0.52	0.49	0.52	0.61	0.56	0.58 (0.09)
Pro	0.34	0.34	0.34	0.28	0.26	0.27	0.30	0.29	0.32	0.33	0.35	0.34	0.31 (0.03)
Lys	0.41	0.41	0.40	0.32	0.29	0.30	0.34	0.33	0.35	0.36	0.40	0.41	0.36 (0.04)
Tyr	0.16	0.15	0.15	0.12	0.10	0.11	0.13	0.13	0.14	0.14	0.16	0.14	0.14 (0.02)
Met	0.17	0.17	0.17	0.13	0.12	0.12	0.13	0.13	0.14	0.14	0.17	0.17	0.14 (0.02)
Val	0.42	0.41	0.40	0.33	0.29	0.31	0.35	0.33	0.36	0.38	0.41	0.41	0.37 (0.04)
Ile	0.30	0.30	0.30	0.24	0.21	0.23	0.25	0.23	0.25	0.27	0.30	0.30	0.27 (0.03)
Leu	0.51	0.52	0.51	0.40	0.36	0.38	0.42	0.40	0.44	0.45	0.52	0.51	0.45 (0.06)
Phe	0.34	0.34	0.34	0.27	0.24	0.26	0.29	0.27	0.30	0.31	0.35	0.34	0.30 (0.04)

### Temporal variation

There were significant temporal patterns in the content of glucose, guluronic acid, proteins, nitrogen, lipids, phosphorous, iodine, arsenic, and mercury, and for the M:G ratio (Tables 4 and 5)*.* The two main sugars, glucose (range: 7.7 – 18.2% DW, *n* = 12) and guluronic acid (range: 8.8 – 13.7% DW, *n* = 12), showed an inverse relationship where guluronic acid peaked in austral spring but was low during austral summer/autumn and glucose peaked in early austral autumn but was low during austral spring (Fig. 3b). The M:G ratio (range: 0.72 – 0.96, *n* = 12) varied with the content of guluronic acid, with the highest in austral summer and lowest in austral winter. Proteins (range: 5.6 – 7.6% DW, *n* = 12) and lipids (range: 0.1—1.6% DW, *n* = 72) showed a unimodal relationship with highest content in late austral spring/early summer and lowest content in austral autumn/winter. Among amino acids, glutamic acid and aspartic acid (range: 1.0 – 1.7% DW and 0.7—1.0% DW, respectively, *n* = 12) were most abundant and accounted for more than 30% of total amino acids on average across a full year (Table 3). The content of nitrogen (range: 1.2 – 1.6% DW, *n* = 72) and phosphorous (range: 0.08 – 0.14% DW, *n* = 12) followed a similar pattern, both being higher in late austral spring/early summer and low in austral autumn/winter. Accordingly, the average nitrogen to protein conversion factor (Protein:N) across all sites and months was 4.9 ± 0.4 SD (*n* = 23), with no significant temporal pattern (Table 5). Iodine, arsenic, and mercury (range: 0.46 – 1.06% DW, 52.5 – 77.4 ppm DW, and 0.01 – 0.03 ppm DW, respectively, *n* = 12) also followed a unimodal pattern with highest content in late austral autumn/early winter and lowest content in late austral spring/early summer. Non-metric MDS showed a clear circular pattern among monthly samples demonstrating strong temporal correlation in biomass composition (Fig. 4b). No significant temporal variability was found for total carbohydrates, mannuronic acid, fucose, mannose, galactose, xylose, phlorotannins, ash, potassium, sulfur, cadmium, or lead (Table 5).

**Table 4:** Temporal variation in biomass content of *E. radiata* collected monthly at Motuotau Island in New Zealand from November 2019 to November 2020 with SD shown in brackets (*n* = 72 for components measured with replication and *n* = 12 for other components). Total carbohydrates (Carbs), phlorotannins (Phlo), proteins, ash, lipids, glucose (Glc), guluronic acid (GulA), mannuronic acid (ManA), fucose (Fuc), mannose (Man), galactose (Gal), xylose (Xyl), nitrogen (N), phosphorus (P), potassium (K), sulfur (S), and iodine (I) are shown as % DW. Arsenic (As), cadmium (Cd), lead (Pb), and mercury (Hg) are shown as ppm DW. M:G shows the mannuronic to guluronic acid ratio, Protein:N shows the nitrogen to protein conversion factor, and C:N shows the carbon to nitrogen ratio

	Nov	Dec	Jan	Feb	Mar	May	Jun	Jul	Aug	Sep	Oct	Nov	Mean
Carbs _(%)_	59.3 (4.2)	58.2 (2.5)	57.5 (4.1)	63.6 (1.5)	63.7 (2.0)	62.8 (1.7)	58 (1.7)	60 (2.8)	60 (4.1)	59.5 (1.5)	58.6 (1.6)	53.8 (4.3)	59.7 (2.9)
Phlo _(%)_	7.9 (2.1)	9.3 (1.1)	8.8 (1.2)	8.5 (1.2)	9.3 (1.1)	8.1 (1.1)	9.0 (1.8)	7.8 (1.1)	8.5 (1.7)	7.6 (1.8)	7.6 (0.5)	9.5 (1.4)	8.5 (0.7)
Protein _(%)_	7.5	7.6	7.5	6.3	5.6	5.6	6.0	6.1	6.3	6.4	7.1	6.9	6.6 (0.7)
Ash _(%)_	23.7 (4.9)	23.4 (1.7)	25.1 (3.2)	20.8 (1.8)	20.8 (1.4)	23.2 (1.3)	26.7 (2.3)	25.9 (1.7)	24.9 (2.8)	26.4 (1.4)	26.1 (1.3)	29.0 (2.9)	24.7 (2.4)
Lipids _(%)_	1.6 (0.3)	1.5 (0.2)	1.1 (0.5)	0.8 (0.2)	0.5 (0.4)	0.3 (0.3)	0.3 (0.1)	0.2 (0.2)	0.3 (0.3)	0.1 (0.2)	0.6 (0.3)	0.8 (0.2)	0.7 (0.5)
Glc _(%)_	9.6	15.0	11.6	16.6	18.2	14.8	11.4	10.1	7.8	8.1	7.7	7.9	11.6 (3.6)
GulA _(%)_	11.3	8.9	9.4	8.8	9.7	10.7	12.2	11.5	13.0	13.7	12.3	11.6	11.1 (1.5)
ManA _(%)_	9.0	8.1	9.1	7.7	8.5	9.0	8.8	8.6	10.1	10.5	9.3	9.6	9.0 (0.8)
Fuc _(%)_	1.5	1.2	1.2	1.1	1.1	1.4	1.3	1.2	1.2	1.6	1.5	1.4	1.3 (0.2)
Man _(%)_	0.8	0.8	0.7	0.7	0.8	0.8	0.9	0.7	0.8	1.0	0.9	0.8	0.8 (0.1)
Gal _(%)_	0.6	0.7	0.6	0.6	0.6	0.6	0.6	0.6	0.5	0.6	0.6	0.6	0.6 (0.0)
Xyl _(%)_	0.3	0.3	0.3	0.3	0.3	0.3	0.3	0.3	0.3	0.4	0.3	0.3	0.3 (0.0)
M:G	0.80	0.91	0.96	0.88	0.88	0.84	0.72	0.74	0.78	0.76	0.76	0.83	0.82 (0.07)
N _(%)_	1.6 (0.2)	1.6 (0.1)	1.6 (0.1)	1.3 (0.1)	1.2 (0.1)	1.2 (0.1)	1.3 (0.1)	1.3 (0.1)	1.3 (0.1)	1.3 (0.1)	1.5 (0.0)	1.5 (0.1)	1.4 (0.1)
P _(%)_	0.12	0.14	0.14	0.11	0.09	0.08	0.09	0.10	0.08	0.09	0.12	0.12	0.11 (0.02)
K _(%)_	3.8	3.4	3.7	3.1	3.1	3.7	4.2	4.1	3.5	3.8	4.0	4.0	3.7 (0.4)
S _(%)_	1.1 (0.1)	1.0 (0.1)	1.0 (0.1)	0.9 (0.0)	1.0 (0.1)	1.0 (0.0)	1.1 (0.1)	1.0 (0.1)	1.1 (0.1)	1.1 (0.1)	1.0 (0.1)	1.1 (0.1)	1.0 (0.1)
Protein:N	4.4	4.9	4.8	4.5	4.6	4.7	4.3	4.7	4.8	4.8	4.7	4.6	4.7 (0.1)
C:N	19.6	20.6	20.6	25.1	26.4	26.3	24.1	24.3	24.2	22.7	20.6	21.0	23 (2.4)
I _(%)_	0.51	0.51	0.55	0.64	0.67	1.02	1.04	1.06	0.76	0.83	0.66	0.46	0.73 (0.22)
As _(ppm)_	52.5	60.9	54.6	53.0	54.1	61.9	67.9	77.4	61.6	56.6	62.1	58.2	60.1 (7.2)
Cd _(ppm)_	1.9	2.0	1.9	1.7	1.7	1.5	2.0	1.4	1.3	1.2	1.5	1.6	1.7 (0.3)
Pb _(ppm)_	0.3	0.1	0.1	0.3	0.0	0.1	0.2	0.1	0.1	0.2	0.1	0.2	0.1 (0.1)
Hg _(ppm)_	0.01	0.01	0.01	0.01	0.02	0.03	0.03	0.02	0.03	0.02	0.02	0.01	0.02 (0.01)

**Table 5:** Durbin-Watson test of autocorrelation testing for significance of temporal patterns (*n* = 12—72). Degree of autocorrelation is expressed by the d-value. Components marked with an asterisk were not normally distributed for their linear model residuals and thus do not meet the requirements of the test. Significant *p*-values are italicised (α = 0.05). M:G shows the mannuronic to guluronic acid ratio, and Protein:N is the nitrogen to protein conversion factor

Component	n	d-value	*p*-value
Carbohydrates*	12	1.77	0.216
Phlorotannins	72	1.57	0.123
Proteins	12	0.51	< *0.001*
Ash	72	1.44	0.076
Lipids	72	0.45	< *0.001*
Glucose	12	1.05	*0.012*
Guluronic Acid	12	1.30	*0.044*
Mannuronic Acid	12	2.11	0.435
Fucose	12	1.56	0.116
Mannose	12	1.95	0.321
Galactose	12	1.95	0.321
Xylose*	12	1.64	0.152
M:G	12	1.27	*0.038*
Nitrogen	72	0.47	< *0.001*
Phosphorus	12	0.83	*0.002*
Potassium	12	1.46	0.082
Sulphur	72	2.66	0.806
Protein:N	12	2.10	0.424
Iodine	12	0.62	< *0.001*
Arsenic*	12	1.30	*0.043*
Cadmium	12	1.92	0.307
Lead	12	2.52	0.728
Mercury	12	1.06	*0.012*

### Spatial vs. temporal variation

The variation between spatial samples was significantly higher than the variation between temporal samples for sulfur, C:N ratio, lead, and mercury (Online resource 4). No other biomass components showed significant difference between spatial and temporal variation, although the standard deviations between sites were higher than the standard deviations between months for most components (Tables 1 and 4).

### Morphology

**Fig. 5:**
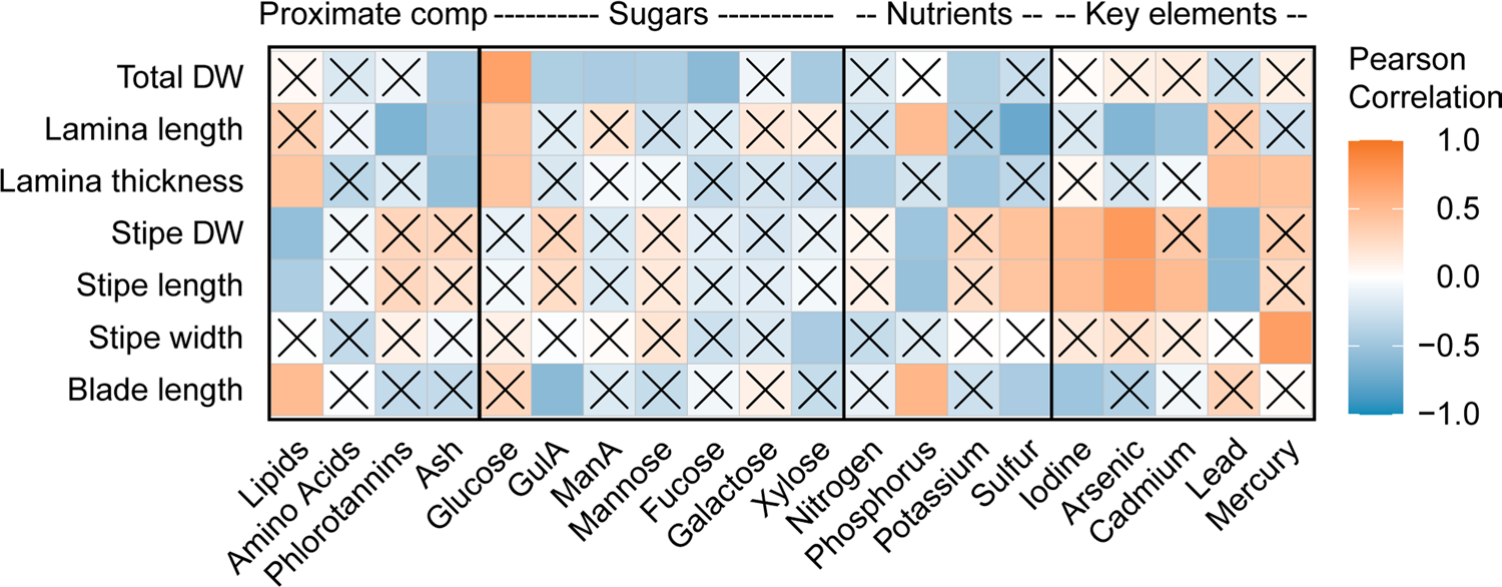
Correlation matrix between morphology and biomass composition of E. radiata (*n* = 23) calculated by Pearson correlation coefficient (r). Orange boxes indicate positive correlation and blue boxes indicate negative correlation. Non-significant correlations are marked with an X (α = 0.05)

Seaweed size was significantly positively correlated with glucose content (linear regression: F(1,21) = 18.12, *p* < 0.001, R^2^ = 0.46, *n* = 23; Fig. 5) but significantly negatively correlated with guluronic acid, mannuronic acid, and fucose content (highest correlation: fucose; linear regression: F(1,21) = 11.01, *p* = 0.003, R^2^ = 0.34, *n* = 23). Phlorotannin content was significantly negatively correlated with lamina length (linear regression: F(1,21) = 15.13, *p* < 0.001, R^2^ = 0.42, *n* = 23). Furthermore, content of sulfur, iodine, and arsenic were all significantly positively correlated with stipe weight (highest correlation: arsenic; linear regression: F(1,21) = 25.49, *p* < 0.001, R^2^ = 0.55, *n* = 23), whereas phosphorus content was significantly positively correlated with blade length (linear regression: F(1,21) = 8.00, p = 0.010, R^2^ = 0.28, *n* = 23). Overall, solids to moisture ratio was high with low variation across replicated samples: 0.20 ± 0.002 SE DW/WW (*n* = 138).

## Discussion

### Biomass composition of E. radiata

*Ecklonia radiata* contains a range of biomass components with commercial potential and may be marketed fresh for human consumption (e.g., Pacific Harvest, New Zealand), processed for extraction of specific components, or fermented for producing biostimulant plant tonics and food products (Battacharyya et al. 2015; Bradly et al. 2021; FAO 2022). In this regard, within the North Island of New Zealand, the major components of *E. radiata* included carbohydrates (mean: 62.4 ± 5.3 SD % DW, *n* = 23), phlorotannins (7.5 ± 2.0 SD % DW, *n* = 138), proteins (6.3 ± 0.9 SD % DW, *n *= 23), and ash (mean: 23.1 ± 4.0 SD % DW, *n* = 138), with minor contributions from lipids (0.8 ± 0.6 SD % DW, *n* = 138).

Mannuronic and guluronic acid (syn. alginate; Szekalska et al. 2016) constituted the majority of the quantified sugars (combined mean: 53.6 ± 7.8 SD mol % of total sugars, equivalent to 20.0 ± 2.1 SD % DW, *n* = 23) and was similar to the commercially cultivated northern hemisphere species *Saccharina latissima* and *Laminaria digitata* (Table 6). The M:G ratio of *E. radiata* was low (range: 0.72 – 1.00, *n* = 23) compared to *E. radiata* from Australia (range: 1.18 – 1.59; Lorbeer et al. 2017) and compared to *S. latissima* and *L. digitata* (Table 6). Alginate with low M:G ratios (high in guluronic acid) forms stronger and more rigid gels, and our results therefore indicate high quality alginate in New Zealand *E. radiata* (McHugh 2003; Porse and Rudolph 2017). Fucose constituted a smaller fraction of the quantified sugars (mean: 4.4 ± 0.7 SD mol % of total sugars, equivalent to 1.3 ± 0.2 SD % DW, *n *= 23), half of that in *S. latissima* and *L. digitata* (Table 6). Assuming a similar composition of fucoidan as in *Ecklonia maxima* (Sichert et al. 2020) the fucose content in *E. radiata* equates to up to 3.9% DW of fucoidan. The content of glucose (a component of laminarin; Stewart et al. 1961) in *E. radiata* biomass (range: 23.9 – 53.9 mol % of total sugars, equivalent to 7.7 – 22.6% DW, *n* = 23) was similar to that of *S. latissima*, but at the lower end of the range recorded for *L. digitata* (Table 6). Overall, the carbohydrate composition *E. radiata* was similar to these commercial northern hemisphere species, although such comparisons may differ between sites (Manns et al. 2017). The inferred levels of alginate, fucoidan, and laminarin (see discussion below) are in accordance with previous findings for *E. radiata* in Australia (Stewart et al. 1961) and contribute significantly to the value of this biomass.

**Table 6:** Biomass composition (% DW) of *E. radiata* and commercial northern hemisphere species *S. latissima* and *L. digitata* with mean values followed by seasonal range in brackets (Manns et al. 2014, 2017; Marinho et al. 2015; Schiener et al. 2015; Sharma et al. 2018). Data estimated from graphs are noted with an asterisk. The seasonal range of laminarin was estimated as the variable seasonal range of glucose minus 2% DW to account for fluctuation of cellulose and is noted with a double asterisk (Black 1950; Stewart et al. 1961)

Component	*E. radiata*	*S. latissima*	*L. digitata*	Reference
Glucose	12.8 (7.7–18.2)	(3–22)*	(6–54.0)*	Manns et al. 2017
Laminarin	(0–9)**	8.2 (1–14)*	6.7 (1–17)*	Schiener et al. 2015
Alginate	20.0 (16.5–24.2)	28.5 (16–31)*	34.6 (16–31)*	Schiener et al. 2015
M:G ratio	0.86 (0.72–0.96)	(1.33–3.53)	(1.47–3.64)	Manns et al. 2017
Fucose	1.3 (1.1–1.6)	2.9	2.4	Manns et al. 2014
Phlorotannins	7.5 (5.2–12.4)	0.41 (0.23–0.68)	0.15 (0.09–0.18)	Schiener et al. 2015
Protein	6.3 (5.6–7.6)	11.0 (5.1–9.9)	6.9 (4.9–8.2)	Schiener et al. 2015
Glutamic acid	1.2 (1.0–1.7)	(1.68–2.97)		Sharma et al. 2018
Aspartic acid	0.9 (0.7–1.0)	(1.40–2.38)		Sharma et al. 2018
Alanine	0.5 (0.5–0.7)	(0.92–1.91)		Sharma et al. 2018
Ash	23.1 (20.8–29.0)	(20–41)*	(11–30)*	Manns et al. 2017
Nitrogen	1.3 (1.1–1.8)	(0.5–3.1)		Marinho et al. 2015
Phosphorous	0.11 (0.08–0.14)	(0.05–0.82)		Marinho et al. 2015

Levels of phlorotannins (range: 3.6 – 12.4% DW, *n* = 138) were tenfold higher than in *S. latissima* and other commercial northern hemisphere species (Schiener et al. 2015; Vilg et al. 2015), but not as high as other sub-tropical browns such as the fucoids *Carpophyllum sp.* and *Cystophora* sp*.* (mean: 13.4% DW and 10.4% DW, respectively; Magnusson et al. 2017). Phlorotannins have substantial commercial potential for cosmetics, functional foods, and biostimulants, given their bioactive activities which includes antioxidant, antimicrobial, antiviral and anti-inflammatory properties (Shrestha et al. 2021).

The content of protein (as total amino acids, range: 4.6 – 7.8% DW, *n *= 23) was similar to *S. latissima* and *L. digitata* (Table 6), with glutamic acid (mean: 15.5 ± 2.2 SD mol % of total amino acids, *n* = 23), aspartic acid (mean: 13.3 ± 0.7 SD mol % of total amino acids, *n* = 23), and alanine (mean: 12.7 ± 0.9 SD mol % of total amino acids, *n* = 23) also being the dominant amino acids as in *S. latissima* (Sharma et al. 2018). Conversely, the maximum nitrogen (mean: 1.3 ± 0.2 SD N % DW, *n* = 138) and phosphorus (mean: 0.11 ± 0.02 P SD % DW, *n* = 23) content of *E. radiata* were approximately twofold and fivefold lower, respectively, than that of northern hemisphere kelp species (Table 6). However, contents of protein and nitrogen were strongly correlated (linear regression: F(1,21) = 259.7, *p* < 0.001, R^2^ = 0.93; Table 5) allowing the calculation of a species specific nitrogen-to-protein conversion factor (Protein:N) for *E. radiata* of 4.9 ± 0.4 SD (*n* = 23), which was not significantly affected by temporal change (Table 5) and very close to the universal seaweed nitrogen-to-protein conversion factor of 4.76 proposed by Angell et al. (2016).

Potentially harmful levels of minerals such as iodine, arsenic, cadmium, lead, and mercury can accumulate in seaweed biomasses, with the risk dependent on the water quality of the growth environment (FAO and WHO 2022; Hahn et al. 2022). Total arsenic contents (mean: 54.5 ± 9.6 SD ppm DW, *n* = 23) exceeded the threshold set by Food Standards Australia New Zealand (FSANZ) of 1 ppm (FSANZ 2019). However, arsenic is only considered toxic to humans in its inorganic form, and the calculated inorganic arsenic (mean: 0.5 ± 0.1 SD ppm DW, *n* = 23, estimated as 1% of total arsenic; Tukai et al. 2002) was twofold below the threshold of 1 ppm. The content of iodine and cadmium in *E. radiata* (mean: 6.3 ± 2.0 SD % DW and 1.4 ± 0.5 SD ppm DW, respectively, *n* = 23) exceeded the recommended threshold values set by the French Agency for Food, Environmental and Occupational Health and Safety (ANSES) for these elements by more than threefold (2000 and 0.5 ppm, respectively), while the content of lead and mercury (mean: 0.2 ± 0.2 SD ppm DW and 0.02 ± 0.01 SD ppm DW, respectively, *n* = 23) were below the threshold (5 and 0.1 ppm, respectively; ANSES 2018, 2020). Cadmium exceeded the recommended threshold level set by ANSES (ANSES 2020), however, to exceed the provisional tolerable intake limit for cadmium (25 µg kg^−1^ bodyweight month^−1^) set by the World Health Organisation (WHO 2011) a 65 kg adult would need to consume 190 g WW *E. radiata* day^−1^. This level of seaweed consumption is considerably higher than the average intake per capita in Japan of 10.4 g seaweed per day (Murai et al. 2021) and is unlikely to occur. Furthermore, blanching is a common processing technique when preparing seaweed for consumption and may reduce iodine content by over 90% (Stévant et al. 2018; Nielsen et al. 2020), which would reduce iodine levels in *E. radiata* below those set by ANSES (2018). Overall, our results indicate that *E. radiata* is suitable for human consumption if consumed within intake limits and/or with appropriate processing.

### Spatial and temporal variation in biomass composition

Spatial and temporal variation in biomass composition can affect the quality of the biomass and therefore have significant implications for the aquaculture of *E. radiata* in New Zealand. Significant spatial variation in the biomass composition of *E. radiata* was detected between sites within the North Island of New Zealand (Table 2). However, no significant differences were found between regions (except for lipids), no clear spatial trends were detected for biomass composition (Fig. 4a), and even sites in close proximity differed substantially in terms of their biomass composition (e.g. Mākara Beach and Titahi Bay: 19 km distance). Consequently, the large variation in biomass composition among sites is likely explained by local rather than regional differences. *Ecklonia radiata* is highly plastic and can express very different morphologies over short distances in response to changing environmental conditions (Fowler-Walker et al. 2006). Changes in lipids, phlorotannins, polysaccharides, and minerals were significantly correlated to stipe, lamina, and blade morphology. Hence, differences in biomass composition may be related to local morphological differences. However, these correlations between morphology and biomass content were mostly relatively weak. Therefore, the large variation in biomass composition between sites is likely caused by other underlying factors such as differences in environmental conditions (e.g. temperature, water chemistry, wave action) and possibly genetic variations (discussed below). For instance, biosynthetic regulation of the ratio between mannuronic and guluronic acid is an essential mechanism in brown algae for controlling strength and flexibility of tissue structure (Indergaard et al. 1990), indicating that alginate composition and abundance may vary between exposed and sheltered sites (Munda 1987; McHugh 2003). Furthermore, nutrient availability is highly variable within the North Island of New Zealand (Plew et al. 2018) which may cause changes in protein and carbohydrate content (Roleda and Hurd 2019). Site-specific environmental conditions thus likely play an important role in the biomass composition of *E. radiata* on the North Island of New Zealand.

Overall, spatial variation among sites was higher than temporal variation between monthly samples for most biomass components (Online resource 4), indicating that geographic variation is higher than temporal variation. Genetic differences between sub-populations could be contributing to the large geographical variation, as the genetic structure of *E. radiata* is strong within the North Island of New Zealand with sharp differentiation between the Wellington region and the other regions, and with further differentiation between most sites at a local level (Nepper-Davidsen et al. 2021). Additionally, differences in biomass components such as phlorotannins and lipids have previously been linked to changes in genotype (Honkanen and Jormalainen 2005; Gosch et al. 2015). However, the strong regional patterns evident in the genetic structure (Nepper-Davidsen et al. 2021) were not reflected in biomass composition here, as the Wellington sites did not stand out from the other sites using clustering analysis (Fig. 4a). To further investigate the matter common-garden experiments (De Villemereuil et al. 2016) are needed.

Significant temporal variation was also evident for several important biomass components of *E. radiata* within the North Island of New Zealand, similar to previous findings for *E. radiata* in Australia (Stewart et al. 1961) and for *S. latissima* and *L. digitata* in northern Europe (Manns et al. 2017). Alginate and fucoidan both peaked in early austral spring, while proteins, lipids, nitrogen and phosphorous peaked in late austral spring/early summer, and total carbohydrates and glucose peaked in early austral autumn. Water temperature is recognised as a key driver of the temporal variation in the biomass composition of seaweeds (compared to nitrogen, phosphorous, and salinity; Manns et al. 2017), and the observed temporal variation in the current study provides further support for strong environmental effects on the composition of *E. radiata* biomass.

Glucose is the main constituent of laminarin—a primary energy storage polysaccharide—and the glucose content of *E. radiata* followed a similar pattern as laminarin in other kelp species, where it builds up during boreal summer and autumn and depletes during boreal winter (Schiener et al. 2015). Furthermore, the temporal changes in glucose content detected in this study (range: 7.7—18.2% DW, *n* = 12) correspond to the temporal change in laminarin content previously found for *E. radiata* in Australia (range: 0.0 – 9.8% DW; Stewart et al. 1961). Glucose is also the main constituent of cellulose—a structural polysaccharide – however, as cellulose content is usually relatively stable throughout the year (1–2% DW yearly fluctuation; Black 1950) the observed temporal changes in glucose content mainly reflect fluctuations in laminarin content.

### Implications for aquaculture

The composition of *E. radiata* biomass was highly site specific and showed large spatial variation in key commercial components, likely related to local differences in environmental conditions. Nepper-Davidsen et al. (2021) recommended not to translocate cultivars of *E. radiata* outside of their area of origin on the North Island of New Zealand to preserve the strong genetic structure of wild populations of this kelp. The biomass composition of local broodstock may therefore be of central importance for optimising the yield of targeted commercial components when choosing sites for seaweed aquaculture. The biomass content of key commercial components also showed significant temporal variation, underlining the importance of harvest timing for optimising yield depending on the biomass components of interest. We suggest early austral spring harvest for high contents of alginate and fucoidan and late austral spring/early summer harvest for proteins and lipids, with the precaution that seasonal patterns may vary between sites (Manns et al. 2017). Total carbohydrates and glucose peaked in early austral autumn, but heavy fouling and high temperatures during austral summer may lower the overall yield and value of the biomass (Handå et al. 2013; Wernberg et al. 2019).

The ratio between carbon and nitrogen content (C:N) in kelp is an indicator of nitrogen availability for primary production, with ratios > 20 indicating nitrogen limited growth (Wernberg et al. 2019). The C:N ratio for E. radiata biomass (mean: 25.8 ± 5.4 SD, *n* = 138) was above 20 during most of the year and at most sites, indicating a high degree of nitrogen limitation both spatially and temporally (Table 1 and 4). Cultivation in areas of high nutrient loads such as those affected by terrestrial runoff or downstream of animal aquaculture could be used to increase growth and simultaneously reduce the effects of nutrient run-off (Handå et al. 2013; Roleda and Hurd 2019). Indeed, based on the nitrogen and phosphorus contents in the biomass, cultivation of *E. radiata* could remove an estimated 164.6 kg N ha^−1^ and 14.7 kg P ha^−1^ per year (see Online resource 5 for calculations).

In conclusion*, E. radiata* had comparable biomass composition to that of commercial northern hemisphere species such as *S. latissima* and *L. digitata* and could be a viable southern hemisphere alternative for a broad range of commercial applications including extraction of phlorotannins, laminarin, and alginate, markets for human and animal consumption, and production of biostimulants.

## Supplementary Information

Below is the link to the electronic supplementary material.

Supplementary file1 (DOCX 33 KB)

## Data Availability

The datasets generated and analysed during the current study are available from the corresponding author on reasonable request.
