# Supplementary material for: High spatial and temporal variation in biomass composition of the novel aquaculture target Ecklonia radiata
Source: J Appl Phycol. 2023 Apr 28:1–14. Online ahead of print. doi: 10.1007/s10811-023-02969-2 (PMC10144899; doi:10.1007/s10811-023-02969-2)
Supplement: Supplementary file 1 — Supplementary file1 (DOCX 33 KB) [file 10811_2023_2969_MOESM1_ESM.docx]

# High spatial and temporal variation in biomass composition of the novel aquaculture target *Ecklonia radiata*

**Journal of Applied Phycology**

**Jacob Nepper-Davidsen^1,2*^, Christopher R. K. Glasson^1,2^, Rebecca J. Lawton^1,2^, Marie Magnusson^1,2^**

^1^Coastal Marine Field Station, School of Science, University of Waikato, Tauranga, New Zealand

^2^Environmental Research Institute, The University of Waikato, Tauranga, New Zealand

Corresponding author email: [Jn100@students.waikato.ac.nz](mailto:Jn100@students.waikato.ac.nz)

**Online resource 1** Morphological data among spatial samples (all sites, top panel, n = 72) and temporal samples (only Motuotau Island, lower panel, n = 72). Stipe width, stipe length, lamina thickness (Lam thick), lamina length (lam length), lamina width (lam width), and blade length are in cm; DW total and DW stipe are in g. Site abbreviations are: Karikari (Kari), Leigh (Leig), Wilson Bay (Wils), Motuotau Island (Motu), Moutohora Island (Mout), Waihau Bay (Waih), Tolaga Bay (Tola), Mahanga (Maha), Māhia (Māhi), Wellington Harbour (WH), Mākara Beach (Māka), and Titahi Bay (Tita)

|  | **Kari** | **Leig** | **Wils** | **Motu** | **Mout** | **Waih** | **Tola** | **Maha** | **M**ā**hi** | **WH** | **M**āka | **Tita** | **Mean** |
| --- | --- | --- | --- | --- | --- | --- | --- | --- | --- | --- | --- | --- | --- |
| **Stipe width** | 0.9 | 1.4 | 2.1 | 1.2 | 1.8 | 1.1 | 1.6 | 1.8 | 1.9 | 1.1 | 1.3 | 1.1 | 1.4 |
| **SD** | (0.1) | (0.4) | (1.1) | (0.1) | (0.2) | (0.4) | (0.1) | (0.2) | (0.1) | (0.2) | (0.2) | (0.1) | (0.4) |
| **Stipe length** | 10.7 | 29.9 | 13.3 | 26.1 | 61.7 | 27.3 | 18.1 | 23.4 | 35.5 | 15.1 | 36.3 | 15.4 | 26.1 |
| **SD** | (4.0) | (15.4) | (4.9) | (20.8) | (12.0) | (11.4) | (7.0) | (6.4) | (10.5) | (3.7) | (13.2) | (2.7) | (13.5) |
| **Lam thick** | 0.3 | 0.3 | 0.4 | 0.3 | 0.5 | 0.4 | 0.8 | 0.9 | 0.9 | 0.4 | 0.4 | 0.4 | 0.5 |
| **SD** | (0.0) | (0.1) | (0.1) | (0.0) | (0.1) | (0.1) | (0.0) | (0.0) | (0.1) | (0.1) | (0.1) | (0.0) | (0.2) |
| **Lam length** | 29.1 | 19.3 | 24.0 | 17.4 | 18.3 | 19.5 | 28.5 | 25.6 | 25.0 | 41.7 | 38.3 | 41.1 | 27.3 |
| **SD** | (5.9) | (5.9) | (6.3) | (5.6) | (4.6) | (6.7) | (6.7) | (6.9) | (4.2) | (4.3) | (14.2) | (9.8) | (8.4) |
| **Lam width** | 6.6 | 7.9 | 8.2 | 5.2 |  |  |  |  |  |  |  |  | 7.0 |
| **SD** | (1.1) | (2.2) | (2.4) | (1.2) |  |  |  |  |  |  |  |  | (1.2) |
| **Blade length** | 28.6 | 33.1 | 40.1 | 33.9 | 32.1 | 34.7 | 36.3 | 30.0 | 37.3 | 29.9 | 31.6 | 30.4 | 33.2 |
| **SD** | (6.0) | (5.4) | (8.6) | (9.8) | (4.3) | (9.3) | (4.1) | (4.5) | (2.3) | (4.4) | (3.7) | (3.2) | (3.3) |
| **DW total** | 54.0 | 78.9 | 55.9 | 46.6 | 155.4 | 50.3 | 88.6 | 66.0 | 95.8 | 77.9 | 79.4 | 120.1 | 80.7 |
| **SD** | (27.0) | (31.8) | (20.5) | (26.7) | (68.8) | (25.0) | (23.9) | (28.7) | (27.3) | (16.0) | (16.2) | (42.8) | (30.4) |
| **DW stipe** | 1.7 | 9.3 | 6.4 | 8.1 | 28.9 | 8.9 | 4.0 | 6.2 | 8.8 | 3.1 | 8.2 | 3.3 | 8.1 |
| **SD** | (1.0) | (5.1) | (4.5) | (8.2) | (12.3) | (5.4) | (1.8) | (2.7) | (3.7) | (1.0) | (3.8) | (1.0) | (6.7) |
|  |  |  |  |  |  |  |  |  |  |  |  |  |  |
|  | **Nov** | **Dec** | **Jan** | **Feb** | **Mar** | **May** | **Jun** | **Jul** | **Aug** | **Sep** | **Oct** | **Nov** | **Mean** |
| **Stipe width** | 1.2 | 1.7 | 1.4 | 2.0 | 1.7 | 1.5 | 1.8 | 1.6 | 1.5 | 1.5 | 1.5 | 1.5 | 1.6 |
| **SD** | (0.1) | (0.2) | (0.2) | (0.2) | (0.1) | (0.1) | (0.2) | (0.1) | (0.2) | (0.1) | (0.3) | (0.2) | (0.2) |
| **Stipe length** | 26.1 | 37.8 | 34.6 | 41.0 | 47.9 | 43.0 | 49.6 | 52.6 | 34.6 | 38.2 | 40.0 | 31.4 | 39.7 |
| **SD** | (20.8) | (10.8) | (16) | (13.6) | (8.1) | (9.3) | (11.8) | (11.7) | (12.4) | (14.5) | (15) | (15.3) | (7.4) |
| **Lam thick** | 0.3 | 0.4 | 0.4 | 1.0 | 0.5 | 0.4 | 0.5 | 0.5 | 0.4 | 0.4 | 0.4 | 0.4 | 0.5 |
| **SD** | (0.0) | (0.0) | (0.1) | (0.1) | (0.1) | (0.1) | (0.1) | (0.1) | (0.1) | (0.1) | (0.1) | (0.1) | (0.2) |
| **Lam length** | 17.4 | 19.8 | 18.7 | 19.6 | 19.8 | 18.7 | 13.2 | 16.2 | 18.5 | 13.6 | 14.7 | 12.7 | 16.9 |
| **SD** | (5.6) | (4.8) | (5.8) | (8.4) | (2.2) | (5.5) | (4.7) | (4.1) | (5.8) | (3.1) | (5.3) | (2.1) | (2.6) |
| **Lam width** | 5.2 |  |  |  |  |  |  | 11.4 | 6.9 | 6.7 | 7.7 | 8.7 | 7.7 |
| **SD** | (1.2) |  |  |  |  |  |  | (1.6) | (2.9) | (1.6) | (2.8) | (2.7) | (1.9) |
| **Blade length** | 33.9 | 37.9 | 38.0 | 38.3 | 19.8 | 28.6 | 27.8 | 26.9 | 18.7 | 24.0 | 27.3 | 28.3 | 29.1 |
| **SD** | (9.8) | (3.6) | (9.7) | (5.4) | (2.2) | (5.8) | (5.6) | (4.2) | (3.2) | (3.4) | (6.8) | (3.9) | (6.4) |
| **DW total** | 46.6 | 128.9 | 92.6 | 117.4 | 134.6 | 81.5 | 76.2 | 80.5 | 46.8 | 42.5 | 62.7 | 54.9 | 80.4 |
| **SD** | (26.7) | (31.3) | (41.9) | (25.1) | (30.0) | (17.8) | (19.2) | (10.8) | (25.0) | (15.5) | (30.1) | (27.9) | (30.9) |
| **DW stipe** | 8.1 | 13.1 | 11.8 | 14.2 | 18.5 | 16.4 | 19.7 | 21.6 | 13.5 | 14.9 | 15.7 | 10.6 | 14.8 |
| **SD** | (8.2) | (6.0) | (8.1) | (6.1) | (1.6) | (3.9) | (4.7) | (6.3) | (10.0) | (8.3) | (9.4) | (6.9) | (3.7) |

**Online resource 2** Elemental analysis across spatial homogenized samples (n = 12) in % DW and ppm. Site abbreviations are: Karikari (Kari), Leigh (Leig), Wilson Bay (Wils), Motuotau Island (Motu), Moutohora Island (Mout), Waihau Bay (Waih), Tolaga Bay (Tola), Mahanga (Maha), Māhia (Māhi), Wellington Harbour (WH), Mākara Beach (Māka), and Titahi Bay (Tita)

|  | **Kari** | **Leig** | **Wils** | **Motu** | **Mout** | **Waih** | **Tola** | **Māha** | **Mahi** | **WH** | **Māka** | **Tita** | **Mean** |
| --- | --- | --- | --- | --- | --- | --- | --- | --- | --- | --- | --- | --- | --- |
| **% DW** |  |  |  |  |  |  |  |  |  |  |  |  |  |
| H | 4.90 | 4.75 | 4.87 | 4.76 | 5.04 | 5.11 | 5.46 | 5.15 | 5.65 | 4.98 | 5.07 | 5.56 | 5.11 |
| C | 32.63 | 31.82 | 31.04 | 31.58 | 32.23 | 32.56 | 34.55 | 32.53 | 34.13 | 31.24 | 31.64 | 34.03 | 32.50 |
| Na | 1.73 | 1.89 | 1.68 | 1.80 | 1.46 | 1.63 | 1.37 | 1.51 | 1.51 | 1.43 | 1.43 | 1.43 | 1.57 |
| Mg | 0.34 | 0.40 | 0.35 | 0.36 | 0.32 | 0.33 | 0.30 | 0.34 | 0.32 | 0.31 | 0.31 | 0.31 | 0.33 |
| Ca | 1.08 | 1.06 | 1.03 | 1.01 | 1.08 | 1.01 | 0.90 | 1.09 | 0.96 | 1.14 | 1.14 | 1.01 | 1.04 |
| **Ppm** |  |  |  |  |  |  |  |  |  |  |  |  |  |
| Al | 26.55 | 5.36 | 15.22 | 5.20 | 3.08 | 8.96 | 10.13 | 5.25 | 5.86 | 2.96 | 3.67 | 13.90 | 8.85 |
| V | 0.77 | 0.67 | 0.33 | 0.61 | 0.82 | 0.69 | 0.35 | 1.03 | 0.67 | 0.65 | 1.23 | 0.78 | 0.72 |
| Cr | 0.30 | 0.19 | 0.17 | 0.15 | 0.23 | 0.43 | 0.17 | 0.17 | 0.16 | 0.19 | 0.12 | 0.17 | 0.21 |
| Mn | 3.11 | 2.88 | 3.04 | 2.93 | 2.05 | 2.17 | 2.22 | 2.20 | 2.49 | 2.88 | 2.23 | 3.03 | 2.60 |
| Fe | 89.94 | 24.01 | 36.16 | 19.82 | 25.41 | 44.14 | 45.65 | 25.82 | 30.82 | 56.40 | 26.21 | 48.28 | 39.39 |
| Co | 0.09 | 0.18 | 0.10 | 0.06 | 0.20 | 0.11 | 0.23 | 0.20 | 0.17 | 0.07 | 0.09 | 0.14 | 0.14 |
| Ni | 0.31 | 0.35 | 0.19 | 0.25 | 0.33 | 0.21 | 0.28 | 0.18 | 0.25 | 0.43 | 0.25 | 0.24 | 0.27 |
| Cu | 0.42 | 0.78 | 0.79 | 0.81 | 0.68 | 1.01 | 1.75 | 0.61 | 1.14 | 7.27 | 0.65 | 1.08 | 1.42 |
| Zn | 3.73 | 5.32 | 6.53 | 8.26 | 4.05 | 4.78 | 6.93 | 5.55 | 6.50 | 10.28 | 3.21 | 7.56 | 6.06 |
| Se | 0.06 | 0.03 | 0.04 | 0.04 | 0.03 | 0.02 | 0.03 | 0.03 | 0.06 | 0.04 | 0.04 | 0.03 | 0.04 |
| Sr | 640.2 | 701.4 | 770.8 | 729.6 | 786.4 | 746.1 | 622.0 | 780.9 | 645.4 | 882.2 | 866.5 | 728.4 | 741.66 |
| Se | 0.09 | 0.05 | 0.05 | 0.07 | 0.06 | 0.04 | 0.05 | 0.04 | 0.05 | 0.08 | 0.05 | 0.06 | 0.06 |
| Ag | 0.19 | 0.86 | 2.23 | 0.62 | 0.81 | 0.85 | 0.14 | 0.90 | 0.73 | 1.34 | 0.48 | 0.14 | 0.77 |
| Ba | 7.74 | 8.16 | 10.44 | 7.90 | 7.40 | 8.98 | 10.60 | 9.18 | 8.29 | 10.35 | 9.23 | 9.10 | 8.95 |
| U | 0.20 | 0.26 | 0.30 | 0.33 | 0.18 | 0.29 | 0.15 | 0.32 | 0.13 | 0.26 | 0.23 | 0.20 | 0.24 |

**Online resource 3** Elemental analysis across temporal homogenized samples collected monthly at Motuotau Island (n = 12) in % DW and ppm

|  | **Nov** | **Dec** | **Jan** | **Feb** | **Mar** | **May** | **Jun** | **Jul** | **Aug** | **Sep** | **Oct** | **Nov** |
| --- | --- | --- | --- | --- | --- | --- | --- | --- | --- | --- | --- | --- |
| **% DW** |  |  |  |  |  |  |  |  |  |  |  |  |
| H | 4.89 | 5.08 | 4.99 | 5.22 | 5.13 | 4.97 | 4.78 | 4.96 | 4.97 | 4.75 | 4.85 | 4.74 |
| C | 31.46 | 32.99 | 32.16 | 33.22 | 32.97 | 31.79 | 30.86 | 31.25 | 31.67 | 30.44 | 30.96 | 30.96 |
| Na | 1.45 | 1.41 | 1.40 | 1.41 | 1.38 | 1.34 | 1.33 | 1.64 | 1.25 | 1.36 | 1.37 | 1.35 |
| Mg | 0.30 | 0.30 | 0.30 | 0.30 | 0.30 | 0.29 | 0.28 | 0.32 | 0.27 | 0.29 | 0.29 | 0.28 |
| Ca | 1.02 | 0.88 | 0.95 | 0.85 | 0.96 | 1.11 | 1.18 | 1.05 | 1.20 | 1.28 | 1.17 | 1.07 |
| **Ppm** |  |  |  |  |  |  |  |  |  |  |  |  |
| Al | 6.09 | 9.01 | 4.04 | 6.31 | 7.84 | 11.12 | 18.98 | 14.70 | 4.54 | 13.35 | 13.25 | 13.92 |
| V | 0.63 | 0.75 | 0.74 | 0.61 | 0.99 | 0.77 | 0.71 | 0.65 | 0.55 | 0.55 | 0.61 | 0.68 |
| Cr | 0.16 | 0.14 | 0.15 | 0.33 | 0.17 | 0.23 | 0.27 | 0.22 | 0.16 | 0.23 | 0.25 | 0.18 |
| Mn | 2.89 | 3.22 | 2.88 | 2.63 | 2.54 | 2.23 | 2.48 | 2.04 | 1.93 | 1.87 | 2.50 | 2.98 |
| Fe | 21.53 | 22.24 | 22.52 | 35.23 | 21.08 | 32.18 | 38.51 | 34.65 | 16.48 | 20.33 | 29.62 | 30.84 |
| Co | 0.06 | 0.08 | 0.07 | 0.09 | 0.09 | 0.10 | 0.12 | 0.13 | 0.08 | 0.07 | 0.07 | 0.07 |
| Ni | 0.26 | 0.34 | 0.24 | 0.33 | 0.29 | 0.33 | 0.41 | 0.40 | 0.24 | 0.24 | 0.27 | 0.21 |
| Cu | 0.81 | 0.84 | 0.92 | 1.16 | 0.82 | 1.09 | 1.12 | 1.46 | 0.64 | 0.81 | 0.80 | 0.68 |
| Zn | 8.57 | 7.05 | 6.02 | 6.06 | 5.64 | 8.00 | 8.94 | 10.26 | 6.49 | 7.64 | 6.94 | 6.63 |
| Se | 0.02 | 0.03 | 0.03 | 0.03 | 0.03 | 0.04 | 0.04 | 0.06 | 0.02 | 0.02 | 0.03 | 0.06 |
| Sr | 774.5 | 569.4 | 650.7 | 540.0 | 611.9 | 779.9 | 872.5 | 988.8 | 931.1 | 1038.0 | 921.1 | 785.07 |
| Se | 0.06 | 0.07 | 0.07 | 0.07 | 0.08 | 0.06 | 0.05 | 0.06 | 0.05 | 0.05 | 0.07 | 0.10 |
| Ag | 0.69 | 0.72 | 0.60 | 0.67 | 0.74 | 0.93 | 0.92 | 1.06 | 0.61 | 0.50 | 0.77 | 0.61 |
| Ba | 7.91 | 4.73 | 7.38 | 4.45 | 5.34 | 7.74 | 8.35 | 10.21 | 8.96 | 9.04 | 8.35 | 8.16 |
| U | 0.31 | 0.21 | 0.25 | 0.20 | 0.26 | 0.21 | 0.23 | 0.25 | 0.33 | 0.39 | 0.29 | 0.38 |

**Online resource 4** Spatial and temporal variation between samples (n = 12). Differences between spatial and temporal variation was analysed using Levene’s test of equal variance (n = 24). Significant p-value are italicised (α = 0.05)

|  | **Spatial**  **% SD of mean** | **Temporal**  **% SD of mean** | **df** | **F-value** | **p-value** |
| --- | --- | --- | --- | --- | --- |
| Lipids _(%)_ | 61.7 | 75.1 | 1,22 | 0.12 | 0.733 |
| Protein _(%)_ | 19.1 | 11.0 | 1,22 | 1.58 | 0.222 |
| Phlo _(%)_ | 23.7 | 8.2 | 1,22 | 3.07 | 0.094 |
| Ash _(%)_ | 15.1 | 9.8 | 1,22 | 1.20 | 0.286 |
| Carbs _(%)_ | 7.6 | 4.8 | 1,22 | 3.04 | 0.095 |
| Glc _(%)_ | 34.4 | 32.1 | 1,22 | 0.11 | 0.740 |
| GulA _(%)_ | 11.2 | 14.5 | 1,22 | 0.56 | 0.462 |
| ManA _(%)_ | 10.4 | 8.8 | 1,22 | 0.36 | 0.557 |
| Fuc _(%)_ | 8.7 | 12.8 | 1,22 | 2.70 | 0.115 |
| Man _(%)_ | 9.7 | 10.3 | 1,22 | 0.11 | 0.746 |
| Gal _(%)_ | 10.3 | 6.0 | 1,22 | 1.81 | 0.192 |
| Xyl _(%)_ | 5.8 | 6.5 | 1,22 | 0.08 | 0.780 |
| M:G | 8.6 | 8.9 | 1,22 | 0.00 | 0.955 |
| N _(%)_ | 18.7 | 10.3 | 1,22 | 1.00 | 0.329 |
| P _(%)_ | 24.3 | 21.0 | 1,22 | 0.18 | 0.674 |
| K _(%)_ | 19.3 | 9.7 | 1,22 | 3.54 | 0.073 |
| S _(%)_ | 15.2 | 5.3 | 1,22 | 7.15 | *0.014* |
| Protein:N | 4.0 | 2.0 | 1,22 | 2.59 | 0.122 |
| C:N | 21.0 | 10.5 | 1,22 | 4.44 | *0.047* |
| I _(%)_ | 23.2 | 29.9 | 1,22 | 2.54 | 0.125 |
| As _(ppm)_ | 16.7 | 11.9 | 1,22 | 0.16 | 0.695 |
| Cd _(ppm)_ | 44.7 | 16.4 | 1,22 | 2.82 | 0.107 |
| Pb _(ppm)_ | 68.7 | 54.8 | 1,22 | 5.70 | *0.026* |
| Hg _(ppm)_ | 72.4 | 33.9 | 1,22 | 10.40 | *<0.001* |

**Online resource 5** Cultivation of *E. radiata* (mean nitrogen: 1.3 ± 0.2 SD % DW, n = 138, and mean phosphorous: 0.11 ± 0.03 SD % DW, n = 23) could remove an estimated 164.6 kg N ha^-1^ and 14.7 kg P ha^-1^ per year. This is assuming a farm layout with 10 rows of 100 m backbone lines ha^-1^ with 10 m deep loops droppers every 1 meter of backbone (20 m seaweed line per loop) and with 8 seaweed specimens on average per meter seaweed line (80 g DW (sample mean) * 10 * 100 * 20 * 8 = 12.8 t DW ha^-1^)
